# Validation of Walking Questionnaire for Population-based Prospective Studies in Japan: Comparison with Pedometer

**DOI:** 10.2188/jea.12.305

**Published:** 2007-11-30

**Authors:** Yoshitaka Tsubono, Ichiro Tsuji, Kazuki Fujita, Naoki Nakaya, Atsushi Hozawa, Takayoshi Ohkubo, Aya Kuwahara, Yoko Watanabe, Keiko Ogawa, Yoshikazu Nishino, Shigeru Hisamichi

**Affiliations:** Division of Epidemiology, Department of Public Health and Forensic Medicine, Tohoku University Graduate School of Medicine.

**Keywords:** physical activity, questionnaire, reproducibility, validity, walking

## Abstract

PURPOSE: To assess the reproducibility and validity of a single-item, self-administered questionnaire on walking used in two population-based prospective cohort studies in northern Japan, using pedometer counts as the reference standard.

METHODS: Fifty-one men and 55 women participating in the main cohort studies (mean age: 61.7 years) responded to a question on the average duration of walking per day five times at 3-month intervals. The subjects also provided 3 consecutive days of pedometer counts four times along with the first four questionnaire surveys.

RESULTS: For the first and the fifth questionnaires administered one year apart, 55% of the subjects chose concordant categories among three options (=<30 min / between 30 and 60 min / >= 60 min), and 13% chose the highest category in one questionnaire and the lowest in the other questionnaire. The sex- and age-adjusted mean daily numbers of walking steps counted by the pedometer were 5,857, 7,047, and 7,621 for the three categories of walking duration in the fifth questionnaire, and it showed significant linear associations with all of the five questionnaire measurements.

CONCLUSON: The single-item questionnaire on walking is reasonably reproducible and valid, and useful in studying the health effects of walking among the Japanese population.

Protective health effects of physical activity have been established.^1^ Recent prospective cohort studies have focused on the effect of walking, one of the most common forms of moderate-intensity physical activity among middle-aged and older populations, and found inverse associations with all cause mortality,^2^^-^^4^ incidence of cardiovasucular disease,^3^ coronary heart disease,^4^^-^^6^ stroke,^7^ type 2 diabetes,^8^ hypertension,^9^ and cancer.^4^

These studies use questionnaires to collect information on regular levels of walking. The self-reports of walking and other forms of physical activity have been validated against physical activity recalls and records,^10^ or against activity records, energy expenditure measured by the accelerometer, and measures of cardiorespiratory fitness and body fatness.^11^ Some studies have not documented the validity of walking questions.^3^^-^^5^^,^^9^

Pedometer directly counts the number of steps walked, and the measurement is independent of errors associated with subjects’ recall or recording of walking. Although pedometer counts may be useful as the reference standard in assessing the validity of questionnaires on walking, only the limited number of studies have been reported.^12^^,^^13^ In this study, we assess the reproducibility and validity of a single-item, self-administered questionnaire on walking used in two population-based prospective cohort studies in northern Japan, using pedometer measurements as the reference standard.

## SUBJECTS AND METHODS

This study was conducted as part of an investigation to assess the reproducibility and validity of a self-administered food frequency questionnaire used in two population-based prospective cohort studies in Miyagi Prefecture in the northern Japan that were started in 1990^14^ and in 1994.^15^ The subjects were participants in one or two of the cohort studies who lived in two rural towns. We selected 59 men and 60 women on the voluntary basis. Written informed consent was obtained from all the subjects.

We conducted five surveys with 3-month intervals from November 1997 through November 1998. During each of the first four surveys, pedometer measurement was conducted for three consecutive days (a total of 12 days), and the self-administered questionnaire including a single-item question on the average duration of walking per day was surveyed on the first day. Only the questionnaire was administered for the fifth survey.

## MEASUREMENTS

The question on walking was worded as “How long do you walk a day on average?” and the subjects were asked to choose one out of three options; 30 minutes or less, between 30 minutes and one hour, and one hour or more.

The subjects were instructed to wear the electronic pedometer (Select II, Suzuken, Nagoya, Japan) from the moment they got up until they went to bed. The pedometer was firmly attached to the clothes at the waist with the aid of a clip. The number of steps counted by the pedometer was read and recorded at the end of each day by the participants. They were instructed to reset the instrument to zero at the end of each day. The reproducibility and validity of the pedometer in counting walking steps were fully evaluated.^16^ We asked subjects to record any problems or incompleteness during the survey (for instance, forgetting to attach the pedometer for part of the day) to subsequently exclude inadequate measurements.

## STATISTICAL ANALYSIS

We excluded 7 subjects providing less than 10 days of adequate pedometer data and 6 subjects who did not responded to all the questionnaires. We used 106 remaining subjects (51 men and 55 women) for this analysis. There were 84, 15, and 7 subjects providing pedometer counts for 12, 11, and 10 days, respectively, with a total of 1,243 person-days of observations.

As a measure of the reproducibility of the questionnaire, we computed the proportions of subjects responding to the concordant categories or the extremely discordant categories (the highest in one questionnaire and the lowest in the other questionnaire) for all combinations of the five questionnaires (10 pairs).

As the reference standard in assessing the validity of the questionnaire, we used the number of walking steps measured by the pedometer that was averaged for each subject over the number of days in which the pedometer counts were available (10-12 days). We compared the geometric mean levels according to the three categories of walking question. The mean values were adjusted for sex and age in years by analysis of covariance using the SAS GLM procedure.^17^ We computed two-sided p-values for linear trend by treating the categories of walking question as an ordinal variable.

## RESULTS

The mean age (standard deviation; SD) of the subjects was 61.7 (8.6) years. Most of the men were engaged in farming or manual labor, and most of the women did farming and/or housekeeping. The mean (SD) levels of body mass index, weight (kg) divided by the square of height (m), were 24.2 (2.9) for men, and 24.9 (3.4) for women.

Table 1 shows the distributions of responses on walking questions. The majority of the subjects chose “1 hour or more”, followed by “between 30 minutes and 1 hour” and “30 minutes or less”. This order of response remained constant for all but the second questionnaires.

**Table 1.** Distribution of subjects according to responses on walking questions (WQs)*

	Period	Duration of walking per day		

		30 min or less	Between 30 min and 1 hour	1 hour or more
WQ1	Nov 1997	17 (16.0)	25 (23.6)	64 (60.4)
WQ2	Feb 1998	29 (27.4)	19 (17.9)	58 (54.7)
WQ3	May 1998	17 (16.0)	24 (22.6)	65 (61.3)
WQ4	Aug 1998	12 (11.3)	21 (19.8)	73 (68.9)
WQ5	Nov 1998	21 (19.8)	27 (25.5)	58 (54.7)

percentages in parentheses.

Table 2 presents the reproducibility of the questionnaire. The proportion of subjects choosing the concordant categories was 55% for the first and the fifth questionnaires administered with one year interval, and it tended to be higher for pairs of the questionnaires administered with shorter intervals. The proportion of subjects choosing the extremely discordant categories was 13% between the first and the fifth questionnaires, and it remains low and relatively constant for all pairs of the questionnaires.

**Table 2.**
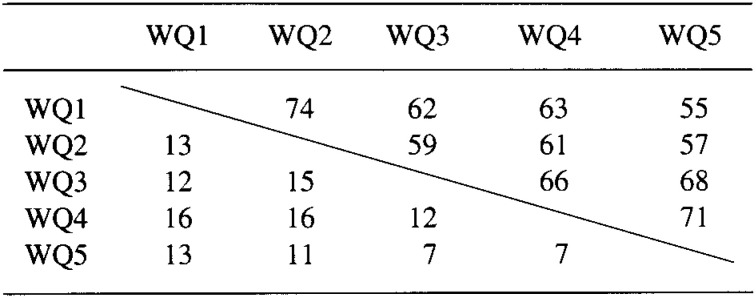
Agreement between walking questionnaires (WQs)*

* Numbers above the diagonal line are the percentages of subjects responding to concordant categories out of three options in pairs of questionnaires. Numbers below are the percentages of subjects responding to extremely discordant categories (i.e., the highest in one questionnaire and the lowest in the other questionnaire).

The mean daily number of walking steps, measured by the pedometer, showed a skewed frequency distribution (Figure). The number of steps per day ranged from 1,116 to 18,936, with the median (inter-quartile range) of 7,286 (4,453). It was slightly higher in men than in women (median = 7,408 and 6,849, respectively) and inversely correlated with age (Spearman r = -0.49).

**Figure.**
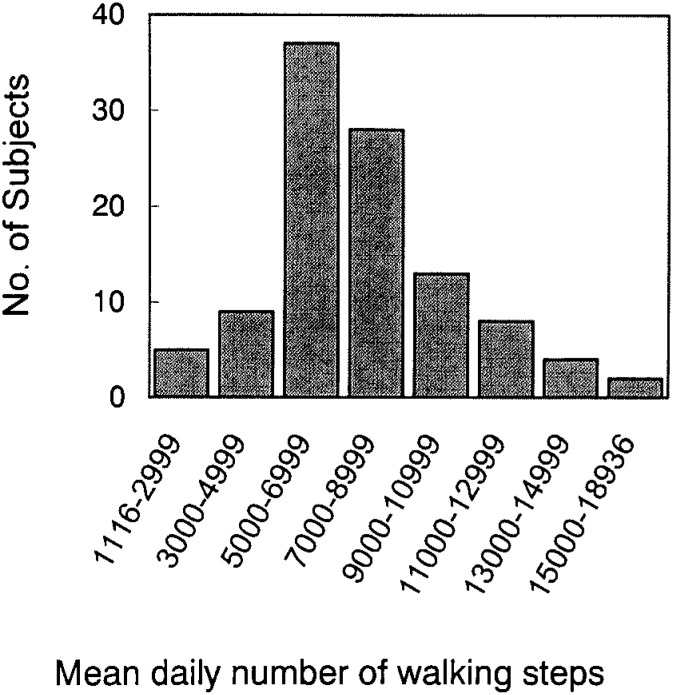
Frequency distribution of the mean daily walking steps measured by pedometer.

Table 3 shows the validity of the questionnaire by comparing the sex- and age-adjusted geometric mean daily number of walking steps according to the three categories of walking questions. The pedometer counts of walking showed a significant linear association with all of the five questionnaire measurements of walking.

**Table 3.** Validity of walking questionnaires (WQs)*

	WQ1	WQ2	WQ3	WQ4	WQ5
30 min or less	5692	6537	5772	5693	5857
Between 30 min and 1 hour	6414	5755	6765	6467	7047
1 hour or more	7820	7903	7610	7548	7621

Trend p	<0.001	0.006	0.003	0.004	0.003

* Numbers are the geometric mean daily number of walking steps counted by the pedometer according to three categories of WQs. The means have been adjusted for sex and age in years by analysis of covariance.

On the same day as the fourth questionnaire was administered, we administered another single-item walking questionnaire used in the national collaborative prospective study of cancer.^18^ This question asked about the average duration of walking outside and inside per day, using four categories. The adjusted geometric mean numbers of walking steps per day for those choosing “hardly walk (n = 4)”, “about 30 minutes (n = 7)” or “30 minutes to 1 hour (n = 11)”, and “1 hour or more (n = 84)” were 6,646, 5,902, and 7,394, respectively (trend p = 0.040).

## DISCUSSION

In this study of a subsample of participants in two population-based prospective cohort studies in northern Japan, a single item question on walking was reasonably reproducible and valid in classifying the subjects according to the actual number of walking steps measured by the pedometer.

This study had several potential limitations. The self-administered questionnaire used in our prospective studies included only two items on physical activities (the average duration of walking per day, and the average duration of engaging in sports and exercise per week). Furthermore, the questionnaire did not ask various ways of walking (inside or outside, at work or at leisure time, etc.) in separate questions. This is because we did not originally intend to calculate total energy expenditure, or quantify the levels of walking according to different ways of walking, based on these questions. Despite the limitation in the original design of our questionnaire, however, it is of notice that even a single question with only three categories of walking is reasonably valid in classifying the subjects according to the relative levels of walking that are measured with pedometer covering all types of walking.

We did not sample the subjects randomly from the participants of main prospective studies but selected them on the voluntary basis. It is probably unlikely, however, that the voluntary nature of the subjects substantially distorted the association between self-reports and pedometer counts of walking.

We recruited the subjects from rural communities in northern Japan. The Japanese National Nutrition Survey^19^ conducted in 1998 using a nationally representative sample reported that the mean daily numbers of walking steps measured by pedometer among subjects who were 60-69 years old were 7,352 for men (n = 830) and 6,907 for women (n = 931). Corresponding values in our subjects of the same age group were 7,519 for men (n = 23) and 6,498 for women (n = 22). Thus, the levels of walking are generally comparable between our subjects and the Japanese general population. Although the majority of our subjects were engaged in farming, many of them did it on the part time basis, so that their levels of walking were not necessarily higher than those of the Japanese general population.

Only a limited number of studies have used pedometer as the reference standard in examining the validity of questionnaires on walking. In a U.S. study of 96 men and women aged 25-70 years examining the validity of the College Alumnus questionnaire,^12^ questionnaire measurement of daily walking distance was moderately correlated with the average of pedometer measurement collected over 7 consecutive days (r= 0.346 for men, and 0.481 for women), but the subjects underestimated their daily walking distance on the questionnaire, compared with the pedometer values. In a study of 493 men and women aged 25-74 years in Switzerland,^13^ self-report of walking and gardening during leisure time was a significant predictor of the average daily number of steps recorded by the pedometer for 7 consecutive days. In a study of 30 elderly men and women aged 63-80 years in the Netherlands,^20^ questionnaire scores of total physical activity were highly correlated with pedometer scores averaged over 3 consecutive days (Spearman r=0.72). However, this study did not report specifically the correlation for walking measured by the questionnaire and the pedometer.

In our study, there should theoretically be at least 30 minutes of difference in time spent for walking per day between subjects choosing the lowest (30 minutes or less) and the highest (one hour or more) categories of the walking question. We actually found approximately 2,000 steps of difference between the two categories (Table 3). Considering that the average number of steps per minute for older Japanese pedestrians at sidewalk is 113 for men and 122 for women,^21^ the 2,000 steps would correspond to 17.7 minutes of walking for men and 16.4 minutes for women. Therefore the questionnaire may overestimate the actual group differences in walking duration. Nevertheless, it should discriminate subjects according to the meaningful contrast of walking, since walking 15-20 min longer per day has been associated with decreased risk of various health outcomes in previous prospective studies.^6^^-^^9^

The reproducibility of our questionnaire decreased over time (Table 2). Both random variations in response to the questionnaire and actual change in walking would contribute to the reduced reproducibility over time. We have observed a similar decrease in the reproducibility of a food frequency questionnaire over 5-year period among the Japanese population.^22^ Although only about a half of subjects (55%) chose the concordant categories for the first and the fifth questionnaires administered with one-year interval, most of the remaining subjects (32%) chose the adjacent categories, and very few subjects (13%) selected the extremely discordant categories. These results indicate that the questionnaire has a modest, but reasonable, degree of reproducibility over one-year interval.

In conclusion, this single-item, self-administered questionnaire on walking used for population-based prospective studies in northern Japan is reasonably reproducible and valid, and useful in studying the health effects of walking among the Japanese population.
